# Innovative Use of Graphite Pencil in Cranioplasty

**DOI:** 10.29252/wjps.9.1.104

**Published:** 2020-01

**Authors:** Padmalakshmi Bharathi Mohan, Ravi Kumar Chittoria, Saurabh Gupta, Abhinav Aggarwal, Chirra Likhitha Reddy, K Shijina, Imran Pathan

**Affiliations:** Department of Plastic Surgery, Jawaharlal Institute of Postgraduate Education and Research (JIPMER), Puducherry, India

**Keywords:** Cranioplasty, Graphite pencil, Methylene blue

**DEAR EDITOR**

Graphite pencil is a common utility used in day to day activity for writing. But, its use has been limited only to writing in paper. In Plastic surgery, marking of the bone or maxillofacial prosthesis is a routine practice, but is usually done with methylene blue or felt tipped skin markers. In this article, we would like to report our innovation of extending the use of graphite pencil in marking cranioplasty moulds. This innovation was done in the Department of Plastic Surgery, in a tertiary care hospital in Puducherry, India. 

During routine cranioplasties done in our department, marking of the mould (acrylic or autologous) was done with methylene blue dye, but faced the disadvantage of getting washed away with irrigation or body fluids. Hence a graphite tip pencil was used in this study. It cost about 0.07 US$. It was sterilized using ethylene trioxide (ETO, Figure 1). It was found that it did not get washed away with irrigation fluids. It did not stain the adjacent tissues and the marking lasted till the end of the procedure Post-procedure, there were no reports of any foreign body reaction or infection.

**Fig. 1:**
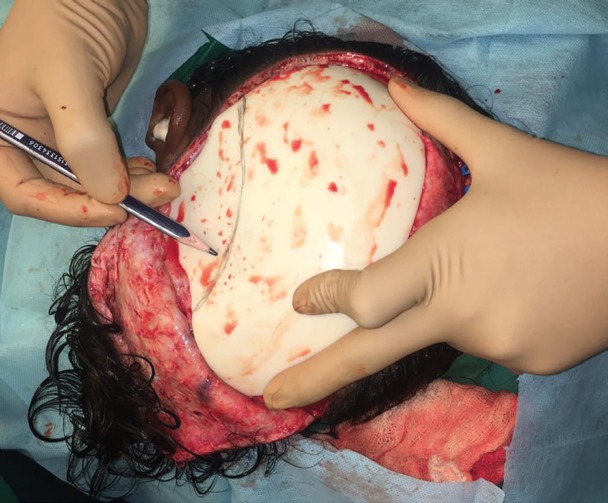
Graphite pencil used in marking of cranioplasty mould

Use of markers in surgeries has been in practice since long time ago. In 1912, the demand for improved skin marking techniques arose when James Thompson popularized the concept of intraoperative mapping for cleft lip repair.^1^ These markings aid the surgeon in presuming the future probable surgical design, before making an irreversible step of the surgery which is cutting. As plastic surgeons, we always use methylene blue, but the markings get obliterated as soon as the procedure begins, due to irrigation fluids and blood. 

Another problem with methylene blue and felt tipped markers is when used in a wet surface, the marking leaks and smudges to the adjacent surfaces. Tattooing is the solution for the above issues; but in bone, it is not possible to tattoo due to absence of dermis. These issues can be easily obviated by the use of graphite pencils. Graphite gets seated in the rough surface of the bone or the polymethylmethacrylate (PMMA) and thus mimics tattooing. Above is also the reason why graphite pencil does not work for smooth implants like stainless steel or non porous titanium.

The advantages of the same are markings did not smudge, when used on wet surfaces and they last longer, even when irrigated or in presence of the blood. The markings can be easily reversed and it can be made accurately.^2^ It is also cost effective and is easily available in any general art store. The sterilization of the same is also simple by means of ETO and can be reused.^3^ One disadvantage is that it cannot be used over smooth implants. Second limitation is that graphite has not been studied for its vitality. (A vital dye gets metabolised by body tissue over time. Methylene blue is a vital dye). It has not been tested for adverse irritable reactions too. 

As discussed by Granick *et al.*,^4^ the ideal surgical marking technique should be non-irritating to the tissues, not permanent when tattooed, and easily removed by the surgeon; however, it should be persistent enough to prevent removal by blood, saline, or other solvents present in the surgical field. Wani *et al.* (2018) introduced the Sharpie markers with ink, which dried on the surface of patient instantaneously and marking did not fade. They did not notice any infection in the patients, and demonstrated that their method was cost effective, and they could use multiple colors.^5^ We successfully showed that graphite pencil fulfilled all the above criteria for marking the tissue.

## CONFLICT OF INTEREST

The authors declare no conflict of interest.
